# Supplementary material for: Spinal cord magnetic resonance imaging and spectroscopy detect early-stage alterations and disease progression in Friedreich ataxia
Source: Brain Commun. 2022 Oct 3;4(5):fcac246. doi: 10.1093/braincomms/fcac246 (PMC9581897; doi:10.1093/braincomms/fcac246)
Supplement: fcac246_Supplementary_Data [file fcac246_supplementary_data.pdf]

Supplementary Materials

Spinal cord MRI and MRS Detect Early-stage Alterations and Disease Progression in Friedreich Ataxia

Table of Contents

Supplementary Figure 1. Comparison of automated segmentation (SCT) with manual segmentation (SpineSeg).

Supplementary Figure 2. Examples of spinal cord MR spectra illustrating spectral quality in 4 different participants with FRDA.

Supplementary Table 1. Comparison of longitudinal results before and after correction of diffusivity values due to the scanner upgrade.

Supplementary Table 2. Comparison of longitudinal results with "All data" and "Same scanner data".

Supplementary Table 3. Additional morphometry data: Cross-sectional and longitudinal results for cross-sectional area (CSA) at individual spinal cord level (C1 to C3) and for grey and white matter.

Supplementary Table 4. Additional DTI data: Cross-sectional and longitudinal results for FA, MD, RD, AD at individual spinal cord levels (C2 to C7).

Supplementary Table 5. Additional MRS data: Longitudinal results for absolute concentrations and for metabolite ratios.

Supplementary Table 6. Additional DTI data: Cross-sectional and longitudinal results for FA, MD, RD, AD in two substructures of the spinal cord [dorsal columns (DC) and cortical-spinal track (CST)] compared to whole cord WM.

Supplementary Table 7. 12-month effect sizes (SRM) from 1-year data only.

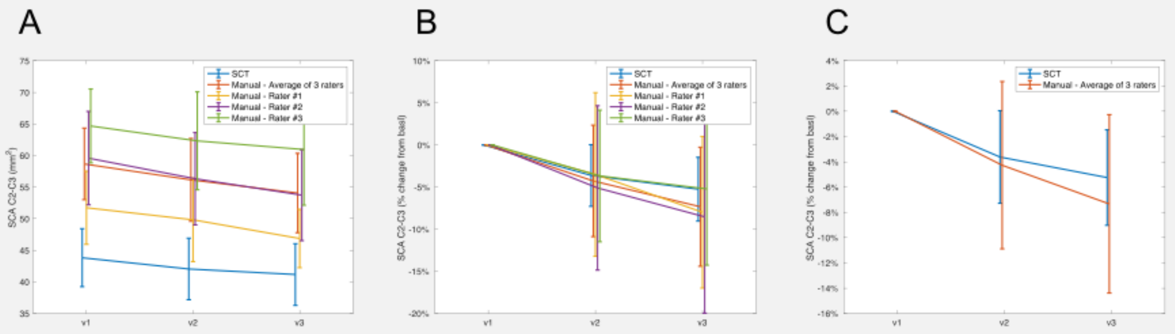

D

	Mean annual slope	SD annual slope	Annual %change from BL	Effect size (SRM)	Raw p-value
CSA (C2-C3) - SCT	-1.03	1.09	-2.4%	-0.95	2E-04
CSA (C2-C3) - Manual - Average of 3 raters	-1.66	1.92	-2.9%	-0.87	7E-04
CSA (C2-C3) - Manual - Rater #1	-1.64	2.35	-3.3%	-0.70	3E-03
CSA (C2-C3) - Manual - Rater #2	-2.31	2.92	-4.0%	-0.79	1E-03
CSA (C2-C3) - Manual - Rater #3	-1.57	2.55	-2.5%	-0.62	8E-03

E

R	SARA	FARS total neuro	Functional staging	ADL	9HPT (non dom.)
CSA (C2-C3) - SCT	-0.55 ***	-0.60 ***	-0.48 ***	-0.51 ***	-0.42 **
CSA (C2-C3) - Manual	-0.61 ***	-0.62 ***	-0.48 ***	-0.48 ***	-0.42 *

Supplementary Figure 1. Comparison of automated segmentation (SCT) with manual segmentation (SpineSeg). *SCT results are the same as in the main manuscript. For comparison, the spinal cord was manually segmented by three separate raters who were blind to disease status and visit number. Datasets were segmented in random order. Each rater segmented the cord on three contiguous slices at the C2-C3 intervertebral disk. Values from all three slices were then averaged to yield a single value of CSA and eccentricity per subject and per rater (A) Mean change in cross-sectional area (CSA) across the 3 visits (B) Percent change in CSA relative to baseline. (C) Same as middle graph after removing curves from individual raters for easier visualization. (D) Comparison of longitudinal metrics (E) Comparison of correlation coefficients.*

All 3 raters found a significant CSA decrease over time, ranging from -2.5% to -4.0%. The annual decrease in CSA for the average of all three raters (-2.9%) was comparable to the annual decrease found with SCT (-2.4%). Effect size was smaller for individual raters (ranging from -0.62 to -0.79) than for the average of all 3 raters (-0.87). Effect size for individual raters was also smaller than for SCT (-0.95), suggesting that automated segmentation with SCT is more precise than manual segmentation.

Note that the absolute value of CSA varied significantly across raters as seen in (A). This is because the determination of the CSF/cord boundary is subjective and very dependent on the contrast and brightness of the computer screen used by each rater. However, the % annual change from baseline was remarkably consistent across raters.

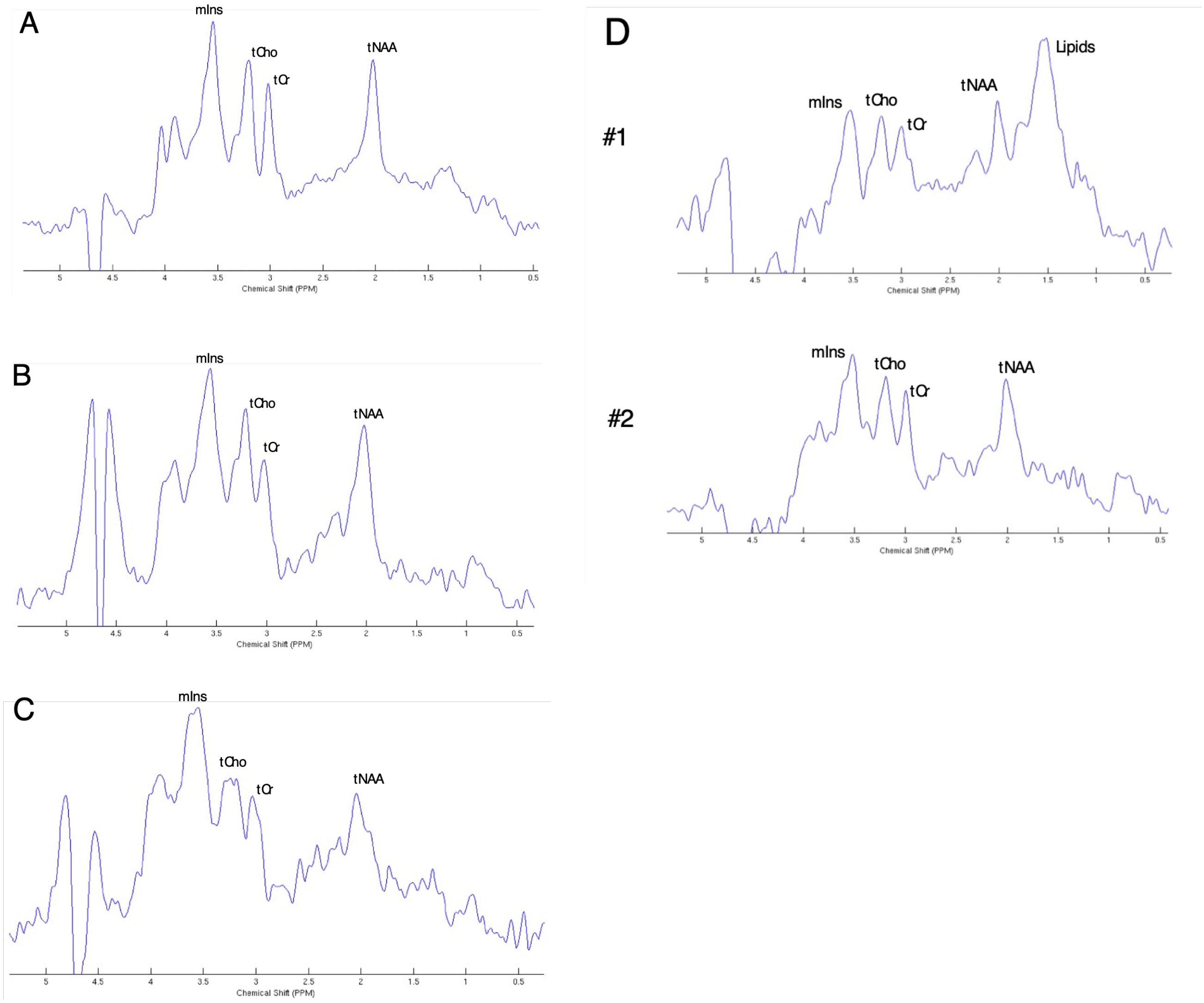

Supplementary Figure 2. Examples of spinal cord MR spectra illustrating spectral quality in 4 different participants with FRDA*. (A) Excellent spectra quality with narrow linewidth (9Hz) and higher SNR (7) (B) Typical spectral quality (LW=16.6Hz, SNR=5) (C) Borderline spectral quality but still usable (LW=21Hz, SNR=3) (D) Two consecutive series (NT=128 each) from the same subject. The first series (#1) was unusable due to high lipid signal. After re-imaging, repositioning the voxel and re-shimming, series #2 was usable (LW=18 Hz, SNR=3), although with lower SNR (NT=128 instead of the full NT=256).*

LW and SNR are values of metabolite linewidth and SNR estimated by LCModel.

Spectra are shown with LB=1Hz and GF=0.1. All spectra are NT=256 except in D as indicated.

Before correction of diffusivity values

	Mean Trio	SD Trio	Mean Prisma	SD Prisma	Diff Prisma vs Trio	Raw p
**CSA (C2-C3) (mm^2)**	70.2	7.5	70.9	7.5	0.9%	0.41
**tNAA/mIns**	1.03	0.16	1.13	0.23	9.3%	0.2
**FA (C3-C6)**	0.54	0.06	0.54	0.04	0.2%	0.96
**MD (C3-C6) (10^{-3} mm^2/s)**	0.97	0.07	1.21	0.08	25.0%	***0.002***
**RD (C3-C6) (10^{-3} mm^2/s)**	0.64	0.09	0.8	0.1	24.1%	***0.009***
**AD (C3-C6) (10^{-3} mm^2/s)**	1.61	0.11	2.03	0.09	25.7%	***0.001***

After correction of diffusivity values (multiplication of Trio values by 1.25)

	mean Trio	SD Trio	mean Prisma	SD Prisma	Diff Prisma vs Trio	Raw p
**CSA (C2-C3) (mm^2)**	70.2	7.5	70.9	7.5	0.9%	0.41
**tNAA/mIns**	1.03	0.16	1.13	0.23	9.3%	0.2
**FA (C3-C6)**	0.54	0.06	0.54	0.04	0.2%	0.96
**MD (C3-C6) (10^{-3} mm^2/s)**	1.21	0.09	1.21	0.08	0.0%	0.99
**RD (C3-C6) (10^{-3} mm^2/s)**	0.8	0.11	0.8	0.1	-0.7%	0.88
**AD (C3-C6) (10^{-3} mm^2/s)**	2.02	0.13	2.03	0.09	0.5%	0.84

Supplementary Table 1. Comparison of longitudinal results before and after correction of diffusivity values due to the scanner upgrade.

Main MR metrics in five healthy volunteers scanned on Trio (VD13D) before the scanner upgrade and on Prisma (VE11C) after the upgrade. Significant differences were found for diffusivity values. A correction factor of 1.25 was computed as the mean ratio of the values MD, RD and AD on Trio vs Prisma over C3-C6. After correction, there was no significant difference in diffusivity values between Trio and Prisma.

All data

Subject	v1	v2	v3
#1	T	T	P
#2	T	T	P
#3	T	T	P
#4	T	T	
#5	T	T	
#6	T	T	P
#7	T	T	P
#8	T	T	P
#9	T	T	P
#10	T	T	P
#11	T	T	P
#12	T	P	P
#13	T	P	P
#14	T	P	P
#15	T	P	P
#16	T	P	P
#17	T	P	P
#18	T	P	P
#19	P	P	P
#20	P	P	P
#21	P	P	P

Same scanner data

Subject	v1	v2	v3
#1	T	T	
#2	T	T	
#3	T	T	
#4	T	T	
#5	T	T	
#6	T	T	
#7	T	T	
#8	T	T	
#9	T	T	
#10	T	T	
#11	T	T	
#12		P	P
#13		P	P
#14		P	P
#15		P	P
#16		P	P
#17		P	P
#18		P	P
#19	P	P	P
#20	P	P	P
#21	P	P	P

All data

	Mean annual slope	SD annual slope	Effect size (SRM)	Annual %change from BL	Raw p
CSA (C2-C3) (mm^2)	-1.03	1.09	-0.95	-2.4%	2E-04
tNAA/mIns	-0.032	0.062	-0.51	-5.8%	0.02
FA (C3-C6)	-0.013	0.026	-0.50	-3.2%	0.02
MD (C3-C6) (10^{-3} mm^2/s)	0.015	0.085	0.18	1.0%	0.22
RD (C3-C6) (10^{-3} mm^2/s)	0.023	0.091	0.25	1.9%	0.14
AD (C3-C6) (10^{-3} mm^2/s)	-0.007	0.103	-0.06	-0.3%	0.39

Same Scanner data

	Mean annual slope	SD annual slope	Effect size (SRM)	Annual %change from BL	Raw p
CSA (C2-C3) (mm^2)	-1.05	1.35	-0.78	-2.4%	1E-03
tNAA/mIns	-0.049	0.091	-0.54	-8.5%	0.02
FA (C3-C6)	-0.020	0.041	-0.48	-4.8%	0.02
MD (C3-C6) (10^{-3} mm^2/s)	0.005	0.108	0.05	0.3%	0.42
RD (C3-C6) (10^{-3} mm^2/s)	0.025	0.118	0.21	2.1%	0.18
AD (C3-C6) (10^{-3} mm^2/s)	-0.046	0.128	-0.36	-2.1%	0.06

Supplementary Table 2. Comparison of longitudinal results with "All data" and "Same scanner data". *To confirm that the scanner upgrade did not bias our results, we performed another analysis with "same scanner data". For any given subject, only data from Trio or data from Prisma were used (whichever number of points was highest). The two tables on the left show the data points used with all data and with same scanner data. The two tables on the right show longitudinal results in each case. The all data table is the same as in the main manuscript (Table 3). The same scanner results are consistent with those obtained with all data.*

Cross-sectional

	Spinal cord level	CTRL	FRDA	Difference (%)	Effect size (Cohen's d)	Raw p
Whole cord (mm^2)	**C1**	65.4 ± 4.7	44.1 ± 5.6	-33%	-4.4	4×10^{-18}
	**C2**	63.2 ± 4.4	43.1 ± 5.3	-32%	-4.5	2×10^{-18}
	**C3**	63.1 ± 4.3	44.2 ± 5.9	-30%	-4.1	6×10^{-16}
	**C1-C2**	64.3 ± 4.4	43.6 ± 5.4	-32%	-4.6	2×10^{-17}
	**C2-C3**	63.2 ± 4.2	43.7 ± 5.5	-31%	-4.4	2×10^{-18}
	**C1-C3**	63.9 ± 4.2	43.8 ± 5.5	-31%	-4.5	5×10^{-18}
White matter (mm^2)	**C1**	54.9 ± 4.3	35.3 ± 5.3	-36%	-4.4	10^{-17}
	**C2**	51.5 ± 3.8	33.6 ± 4.9	-35%	-4.4	10^{-17}
	**C3**	49.8 ± 4.0	33.3 ± 5.3	-33%	-3.8	4×10^{-15}
	**C1-C2**	53.2 ± 4.0	34.5 ± 5.1	-35%	-4.5	3×10^{-11}
	**C2-C3**	50.6 ± 3.8	33.5 ± 5.0	-34%	-4.2	5×10^{-18}
	**C1-C3**	52.3 ± 4.0	34.3 ± 5.1	-34%	-4.3	10^{-16}
Grey matter (mm^2)	**C1**	10.8 ± 0.6	9.1 ± 0.6	-15%	-2.6	6×10^{-12}
	**C2**	11.6 ± 0.5	9.7 ± 0.7	-16%	-3.2	10^{-13}
	**C3**	13.2 ± 0.6	11.3 ± 0.9	-14%	-2.5	6×10^{-11}
	**C1-C2**	11.2 ± 0.5	9.4 ± 0.6	-16%	-3.1	4×10^{-14}
	**C2-C3**	12.4 ± 0.5	10.6 ± 0.7	-15%	-3.0	9×10^{-14}
	**C1-C3**	12.0 ± 0.5	10.2 ± 0.6	-15%	-3.0	5×10^{-14}

Longitudinal

	Spinal cord level	Mean annual slope	SD annual slope	Annual %change from BL	Effect size (SRM)	Raw p
Whole cord (mm^2)	**C1**	-0.96	0.85	-2.2%	-1.13	4×10^{-5}
	**C2**	-1.03	0.96	-2.4%	-1.07	6×10^{-5}
	**C3**	-1.02	1.32	-2.4%	-0.78	10^{-3}
	**C1-C2**	-1.00	0.81	-2.3%	-1.23	10^{-5}
	**C2-C3**	-1.03	1.09	-2.4%	-0.95	2×10^{-4}
	**C1-C3**	-1.00	0.93	-2.3%	-1.09	6×10^{-5}
White matter (mm^2)	**C1**	-0.81	0.93	-2.3%	-0.87	0.001
	**C2**	-1.21	1.02	-3.7%	-1.18	4×10^{-5}
	**C3**	-1.13	1.42	-3.5%	-0.80	0.002
	**C1-C2**	-2.01	1.87	-3.0%	-1.07	0.0001
	**C2-C3**	-1.17	1.15	-3.6%	-1.02	0.0002
	**C1-C3**	-1.94	2.24	-2.9%	-0.87	0.001
Grey matter (mm^2)	**C1**	-0.04	0.37	-0.4%	-0.11	0.6
	**C2**	0.15	0.36	1.6%	0.43	0.1
	**C3**	-0.12	0.45	-1.0%	-0.26	0.3
	**C1-C2**	0.11	0.58	0.6%	0.19	0.4
	**C2-C3**	0.01	0.35	0.1%	0.03	0.9
	**C1-C3**	-0.16	0.52	-0.8%	-0.30	0.2

Supplementary Table 3. Additional morphometry data: Cross-sectional and longitudinal results for cross-sectional area (CSA) at individual spinal cord levels (C1 to C3) and for grey matter and white matter. *Results were obtained from brain T1 images using Spinal Cord Toolbox. Annual slopes were obtained by fitting all 2-year data (3 time points per subject: baseline, 1-year and 2-year follow-up).*

Cross-sectional

	CTRL	FRDA	Difference (%)	Effect size (Cohen's d)	Raw p
**FA (C2)**	0.38 ± 0.15	0.30 ± 0.17	-21%	-0.4	0.2
**FA (C3)**	0.53 ± 0.09	0.40 ± 0.08	-25%	-1.7	3×10^{-6}
**FA (C4)**	0.56 ± 0.08	0.42 ± 0.07	-25%	-1.9	2×10^{-7}
**FA (C5)**	0.54 ± 0.05	0.40 ± 0.05	-25%	-3.0	3×10^{-11}
**FA (C6)**	0.50 ± 0.06	0.39 ± 0.05	-22%	-1.9	10^{-7}
**FA (C7)**	0.46 ± 0.11	0.36 ± 0.06	-22%	-1.3	3×10^{-4}
**FA (C4-C5)**	0.55 ± 0.06	0.41 ± 0.06	-25%	-2.5	4×10^{-10}
**FA (C3-C6)**	0.53 ± 0.06	0.40 ± 0.05	-24%	-2.6	3×10^{-10}
**MD (C2)**	0.85 ± 0.32	1.16 ± 0.54	36%	0.8	0.04
**MD (C3)**	1.17 ± 0.20	1.61 ± 0.35	37%	1.6	2×10^{-5}
**MD (C4)**	1.12 ± 0.21	1.50 ± 0.22	34%	1.8	4×10^{-7}
**MD (C5)**	1.17 ± 0.19	1.63 ± 0.35	39%	2.2	10^{-5}
**MD (C6)**	1.23 ± 0.20	1.58 ± 0.15	29%	2.0	5×10^{-8}
**MD (C7)**	1.24 ± 0.23	1.59 ± 0.28	28%	1.2	2×10^{-4}
**MD (C4-C5)**	1.14 ± 0.19	1.56 ± 0.24	36%	2.1	2×10^{-7}
**MD (C3-C6)**	1.17 ± 0.18	1.58 ± 0.20	35%	2.3	7×10^{-9}
**RD (C2)**	0.58 ± 0.25	0.90 ± 0.43	55%	1.0	0.01
**RD (C3)**	0.80 ± 0.21	1.28 ± 0.38	60%	1.7	8×10^{-6}
**RD (C4)**	0.73 ± 0.20	1.15 ± 0.25	57%	1.9	3×10^{-7}
**RD (C5)**	0.78 ± 0.15	1.28 ± 0.35	63%	2.8	2×10^{-6}
**RD (C6)**	0.85 ± 0.17	1.24 ± 0.15	46%	2.5	10^{-9}
**RD (C7)**	0.89 ± 0.23	1.28 ± 0.26	43%	1.4	3×10^{-5}
**RD (C4-C5)**	0.76 ± 0.16	1.21 ± 0.26	61%	2.5	3×10^{-8}
**RD (C3-C6)**	0.79 ± 0.15	1.24 ± 0.21	56%	2.8	6×10^{-10}
**AD (C2)**	1.39 ± 0.52	1.67 ± 0.79	20%	0.5	0.2
**AD (C3)**	1.92 ± 0.28	2.27 ± 0.34	18%	1.1	0.003
**AD (C4)**	1.90 ± 0.29	2.19 ± 0.19	15%	1.1	2×10^{-4}
**AD (C5)**	1.94 ± 0.29	2.32 ± 0.34	19%	1.2	8×10^{-4}
**AD (C6)**	1.97 ± 0.29	2.26 ± 0.17	14%	1.1	3×10^{-4}
**AD (C7)**	1.95 ± 0.31	2.23 ± 0.35	15%	0.7	0.01
**AD (C4-C5)**	1.92 ± 0.29	2.25 ± 0.22	17%	1.2	10^{-4}
**AD (C3-C6)**	1.93 ± 0.28	2.26 ± 0.19	17%	1.3	4×10^{-5}

Longitudinal

	Mean annual slope	SD annual slope	Annual % change from baseline	Effect size (SRM)	Raw p
**FA (C2)**	-0.042	0.131	-18.1%	-0.3	0.1
**FA (C3)**	0.002	0.042	0.6%	0.1	0.4
**FA (C4)**	-0.028	0.030	-6.4%	-0.9	0.0003
**FA (C5)**	-0.018	0.026	-4.6%	-0.7	0.002
**FA (C6)**	-0.008	0.047	-2.1%	-0.2	0.2
**FA (C7)**	-0.005	0.062	-1.5%	-0.1	0.4
**FA (C4-C5)**	-0.023	0.024	-5.6%	-1.0	0.0002
**FA (C3-C6)**	-0.013	0.026	-3.2%	-0.5	0.02
**MD (C2)**	-0.136	0.474	-15.8%	-0.3	0.1
**MD (C3)**	-0.010	0.175	-0.7%	-0.1	0.4
**MD (C4)**	0.036	0.089	2.5%	0.4	0.04
**MD (C5)**	0.002	0.102	0.1%	0.0	0.5
**MD (C6)**	0.025	0.125	1.6%	0.2	0.2
**MD (C7)**	0.081	0.142	5.0%	0.6	0.01
**MD (C4-C5)**	0.024	0.082	1.6%	0.3	0.1
**MD (C3-C6)**	0.015	0.085	1.0%	0.2	0.2
**RD (C2)**	-0.107	0.367	-15.9%	-0.3	0.1
**RD (C3)**	-0.013	0.163	-1.1%	-0.1	0.4
**RD (C4)**	0.054	0.091	4.9%	0.6	0.01
**RD (C5)**	0.025	0.100	2.1%	0.2	0.1
**RD (C6)**	0.027	0.146	2.1%	0.2	0.2
**RD (C7)**	0.058	0.184	4.5%	0.3	0.1
**RD (C4-C5)**	0.039	0.085	3.4%	0.5	0.03
**RD (C3-C6)**	0.023	0.091	1.9%	0.3	0.1
**AD (C2)**	-0.193	0.692	-15.7%	-0.3	0.1
**AD (C3)**	-0.005	0.223	-0.2%	0.0	0.5
**AD (C4)**	-0.003	0.117	-0.1%	0.0	0.5
**AD (C5)**	-0.039	0.134	-1.8%	-0.3	0.1
**AD (C6)**	0.008	0.141	0.4%	0.1	0.4
**AD (C7)**	0.073	0.176	3.3%	0.4	0.04
**AD (C4-C5)**	-0.014	0.103	-0.7%	-0.1	0.3
**AD (C3-C6)**	-0.007	0.103	-0.3%	-0.1	0.4

Supplementary Table 4. Additional DTI data: Cross-sectional and longitudinal results for FA, MD, RD, AD at individual levels of the spinal cord (C2 to C7). *Unit for MD, RD and AD is 10-3 mm2/s. Annual slopes were obtained by fitting all 2-year data (3 time points per subject: baseline, 1-year and 2-year follow-up).*

	Mean annual slope	SD annual slope	Annual % change from baseline	Effect size (SRM)	Raw p
**tNAA/mIns**	-0.03	0.06	-5.8%	-0.5	0.02
**tNAA (mM)**	-0.21	0.88	-3.9%	-0.2	0.15
**mIns (mM)**	0.07	1.17	0.7%	0.1	0.40
**tCr (mM)**	-0.03	0.79	-0.6%	-0.04	0.43
**tCho (mM)**	-0.03	0.37	-1.3%	-0.1	0.36
**tNAA/tCr**	-0.03	0.17	-2.5%	-0.2	0.24
**mIns/tCr**	0.03	0.21	1.5%	0.1	0.26
**tNAA/tCho**	-0.07	0.43	-2.7%	-0.2	0.25
**mIns/tCho**	0.11	0.65	2.3%	0.2	0.24

Supplementary Table 5. Additional MRS data: Longitudinal results for absolute concentrations and for metabolite ratios*. Annual slopes were obtained by fitting all 2-year data (3 time points per subject: baseline, 1-year and 2-year follow-up).*

Cross-sectional

		CTRL	FRDA	Difference (%)	Effect size (Cohen's d)	Raw p
Whole cord WM (C3-C6)	FA	0.53 ± 0.06	0.40 ± 0.05	-24%	-2.6	3E-10
	MD (10^{-3} mm^2/s)	1.17 ± 0.18	1.58 ± 0.20	35%	2.3	7E-09
	RD (10^{-3} mm^2/s)	0.79 ± 0.15	1.24 ± 0.21	56%	2.8	6E-10
	AD (10^{-3} mm^2/s)	1.93 ± 0.28	2.26 ± 0.19	17%	1.3	4E-05
Dorsal columns (C3-C6)	FA	0.61 ± 0.06	0.44 ± 0.03	-28%	-3.7	2E-15
	MD (10^{-3} mm^2/s)	1.07 ± 0.13	1.47 ± 0.20	37%	2.2	1E-08
	RD (10^{-3} mm^2/s)	0.65 ± 0.09	1.09 ± 0.16	69%	3.4	4E-13
	AD (10^{-3} mm^2/s)	1.92 ± 0.28	2.22 ± 0.35	15%	0.9	7E-03
Cortico-spinal tract (C3-C6)	FA	0.55 ± 0.05	0.43 ± 0.06	-22%	-2.4	2E-08
	MD (10^{-3} mm^2/s)	1.14 ± 0.16	1.48 ± 0.21	30%	2.1	6E-07
	RD (10^{-3} mm^2/s)	0.76 ± 0.13	1.13 ± 0.23	49%	2.6	1E-07
	AD (10^{-3} mm^2/s)	1.91 ± 0.26	2.18 ± 0.20	14%	1.1	4E-04

Longitudinal

		Mean annual slope	SD annual slope	Effect size (SRM)	Annual % change from baseline	Raw p
Whole cord WM (C3-C6)	FA	-1E-02	3E-02	-0.50	-3.2%	0.02
	MD (10^{-3} mm^2/s)	2E-05	9E-05	0.18	1.0%	0.22
	RD (10^{-3} mm^2/s)	2E-05	9E-05	0.25	1.9%	0.14
	AD (10^{-3} mm^2/s)	-7E-06	1E-04	-0.06	-0.3%	0.39
Dorsal columns (C3-C6)	FA	-9E-03	2E-02	-0.39	-2.2%	0.09
	MD (10^{-3} mm^2/s)	-2E-05	1E-04	-0.15	-1.2%	0.51
	RD (10^{-3} mm^2/s)	-2E-06	1E-04	-0.02	-0.2%	0.92
	AD (10^{-3} mm^2/s)	-5E-05	2E-04	-0.26	-2.5%	0.26
Cortico-spinal tract (C3-C6)	FA	-1E-02	3E-02	-0.52	-3.3%	0.03
	MD (10^{-3} mm^2/s)	2E-05	9E-05	0.20	1.2%	0.37
	RD (10^{-3} mm^2/s)	3E-05	9E-05	0.29	2.4%	0.22
	AD (10^{-3} mm^2/s)	-3E-06	1E-04	-0.03	-0.1%	0.91

Supplementary Table 6. Additional DTI data: Cross-sectional and longitudinal results for two substructures of the spinal cord [dorsal columns (DC) and cortical-spinal track (CST)] compared to whole cord WM. *Dorsal columns showed better cross-sectional effect size (Cohen's d) than whole cord WM for FA (-3.7 vs -2.6) and RD (3.4 vs 2.8), but longitudinal effect size and annual change from baseline were more pronounced in whole cord WM. The cortico-spinal tract showed longitudinal changes from baseline and SRM comparable to whole cord WM. Annual slopes were obtained by fitting all 2-year data (3 time points per subject: baseline, 1-year and 2-year follow-up).*

	mean diff 12mo	SD diff 12mo	SRM diff 12mo	%change from BL 12mo	Raw p
**CSA (C2-C3) (mm^2)**	-0.98	1.38	-0.71	-2.2%	6×10^{-5}
**tNAA/mIns**	-0.04	0.09	-0.41	-4.8%	0.01
**FA (C3-C6)**	-0.02	0.05	-0.35	-4.0%	0.02
**MD (C3-C6) (10^{-3} mm^2/s)**	0.018	0.123	0.15	1.4%	0.19
**RD (C3-C6) (10^{-3} mm^2/s)**	0.029	0.127	0.22	2.5%	0.09
**AD (C3-C6) (10^{-3} mm^2/s)**	-0.009	0.167	-0.05	-0.1%	0.37
**SARA**	2.1	1.5	1.45		8×10^{-9}
**FARS total neuro**	5.2	4.5	1.14		5×10^{-9}
**Functional**	0.4	0.5	0.87		10^{-6}
**ADL**	1.9	1.7	1.11		9×10^{-9}
**9HPT (non-dom) (s)**	3.5	7.0	0.50		10^{-3}

Supplementary Table 7. 12-month effect sizes (SRM) from 1-year data only. *Annual slopes were obtained by fitting 1-year data (2 time points per subject: baseline and 1-year follow-up).*
